# Molecular Anatomy and Number of Antigen Specific CD8 T Cells Required to Cause Type 1 Diabetes

**DOI:** 10.1371/journal.ppat.1003044

**Published:** 2012-11-29

**Authors:** Michael B. A. Oldstone, Kurt H. Edelmann, Dorian B. McGavern, Justin T. Cruite, Megan J. Welch

**Affiliations:** Viral-Immunobiology Laboratory, Department of Immunology and Microbial Science, The Scripps Research Institute, La Jolla, California, United States of America; Vanderbilt University Medical Center, United States of America

## Abstract

We quantified CD8 T cells needed to cause type 1 diabetes and studied the anatomy of the CD8 T cell/beta (β) cell interaction at the immunologic synapse. We used a transgenic model, in situ tetramer staining to distinguish antigen specific CD8 T cells from total T cells infiltrating islets and a variety of viral mutants selected for functional deletion(s) of various CD8 T cell epitopes. Twenty percent of CD8 T cells in the spleen were specific for all immunodominant and subdominant viral glycoprotein (GP) epitopes. CTLs to the immunodominant LCMV GP33-41 epitope accounted for 63% of the total (12.5% of tetramers). In situ hybridization analysis demonstrated only 1 to 2% of total infiltrating CD8 T cells were specific for GP33 CD8 T cell epitope, yet diabetes occurred in 94% of mice. The immunologic synapse between GP33 CD8 CTL and β cell contained LFA-1 and perforin. Silencing both immunodominant epitopes (GP33, GP276–286) in the infecting virus led to a four-fold reduction in viral specific CD8 CTL responses, negligible lymphocyte infiltration into islets and absence of diabetes.

## Author Summary

Insulin-dependent type 1 diabetes (T1D) is characterized by elevated blood sugar, lymphocytic infiltration into the islets of Langerhans and T cell destruction of beta (β) cells. β cells produce insulin whose function is to maintain and regulate glucose hemostasis. However, in vivo, the numbers of antigen specific T cells that migrate to the islets to cause T1D, the engagement of such T cells with β cells at the immunologic synapse and the molecules expressed at the synapse are not clear. Using a transgenic model of virus induced T1D, a panel of viruses with CD8 T cell epitope mutations and in situ tetramer hybridization, we note of the total CD8 T cells infiltrating the islets, only 1–2% are antigen specific recognizing the immunodominant virus CD8 T cell epitope expressed on β cells. Immunohistochemical analysis of the synapse found between antigen specific CD8 T cells and β cells displays attachment by LFA-1 and presence of perforin, the molecule indicative of lytic activity.

## Introduction

Insulin-dependent diabetes mellitus, type 1 (T1D), embodies a clinical-pathologic scenario in which numerous beta (β) cells located in the pancreatic islets of Langerhans are destroyed so that insufficient insulin is produced to maintain host glucose homeostasis. This lack of insulin leads to elevated blood glucose levels, which if unchecked cause ketoacidosis resulting in death. T1D unfolds in two steps: first, the initiating event(s) triggers the appropriate T cell immune response; second, that response evokes effector molecules and mechanisms of action that destroy β cells. Initiating events revolve around a host's genes that determine susceptibility and environmental factors such as viruses. In fact, viral infections are repeatedly associated with the onset of T1D in humans [1]–[9] and in animal models [10]–[13]. The second step includes the effector cells and molecules involved in β cell destruction. Although incompletely understood, the cause of β cell damage in T1D has been attributed to the host's own immune response. Information based on biopsied or autopsied pancreases from humans [14]–[17] and study of relevant animal models (reviewed [18] has identified numerous effector cells such as CD8 cytotoxic T cells (CTL), CD4 T cells, macrophages, B cells and NK cells in the islets. Other players in this action are cytokine/chemokines like IFN-γ, TNF-α, CXCR3, CXCL9, CXCL10 (IP-10), and CXCL11 [19]–[21]. However, studies of humans with T1D [14]–[17] indicate that, among many potential effector cells, CD8 CTL predominate. Usually, more than 50% of the cells infiltrating pancreatic islets are CD8 CTL, and these are found at their β cell targets in association with an abundant expression of MHC class I molecules [15], [17], [22]. However, still unknown is what subpopulation and how many CTL specifically recognize the antigen(s) targeted in β cells causing their damage and inducing T1D. A confounding factor is the numerous bystander T cells attracted to the islets by chemokine/cytokine signal(s) and determining what role, if any, they play in the causation of T1D.

Our early studies used limiting dilution analysis of spleens from Balb/c RIP LCMV nucleoprotein (NP) transgenic (tg) mice and infection with a variety of LCMV strains (Armstrong [ARM], E350, Pasteur, Traub) that did or did not cause T1D. Results indicated that one effector virus-specific CD8 T cell per 785–1000 total CD8 T cells was required to cause diabetes [23]. By contrast, ratio of 1/6000 or less failed to cause disease [23]. These results were confirmed studying the role of cytokines/chemokines in the RIP LCMV-NP tg model [20] as one specific CD8 T cell per 1000 total T cells were required to cause T1D. However, the RIP LCMV-NP model is limited by having only one known CTL epitope, NP 118–127 [12], [24], [25], a lack of T cell receptor (TCR) tg mice, other genetically modified mice and reagents that are available for H-2b mice. Thus, in this report we switched to using RIP LCMV glycoprotein (GP) tg H-2b mice which express all four known GP CD8 T cell epitopes: immunodominant GP 33–41>GP 276–286 and subdominant GP 92–101 and GP 118–125. Utilizing LCMV ARM wt or viral variants of LCMV ARM in which CD8 CTL epitopes were mutated [26], [27] along with GFP-labeled TCR GP33 CTLs and tetramers allowed us for the first time to identify the numbers of virus-specific CD8 T cells from the total T cells in the islet target tissue. Further, this technology allowed us to determine the immunologic synapse between the viral-specific CD8 T cell and the β cell target. In both the RIP LCMV-NP and LCMV-GP tg models, when the viral transgene is expressed in the β cell and not in the thymus, T1D is caused exclusively by LCMV-specific CD8 T cells and does not require CD4 T cell help [12], [28].

Here we report quantification and identification of the antigen-specific CD8 CTLs that caused diabetes. Further, we distinguished such antigen-specific cells from the bystander non-antigen-specific CD8 T cells in the islets. Lastly, we directly view the anatomy of antigen-specific CD8 T cell interaction with β cell and record the presence of host-effector molecules at the immunologic synapse between these cells.

## Results

### T1D in RIP-LCMV GP tg mice infected with LCMV or LCMV GP variants: Quantification of the virus-specific CTL response

**Figure 1:**
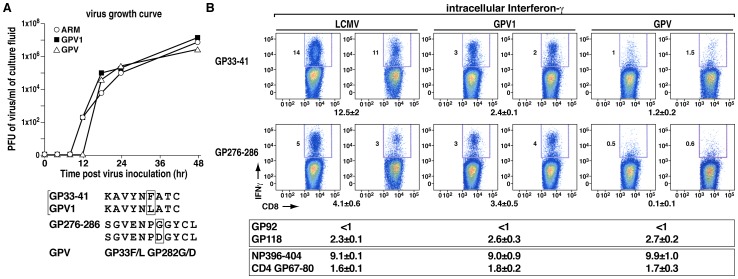
Growth of wt LCMV ARM and its two variants GPV1, GPV; and generation of intracellular IFN-γ in LCMV-specific T cells from the spleens of C57Bl/6 mice 7 days after viral infection. The GP LCMV variants were made as described in Materials and Methods [26], [27]. The GP 33–41 and GP 276–286 immunodominant CTL epitopes of GPV are non-functional, whereas the subdominant CTL epitopes GP 92–101 and GP 118–125 are fully functional. GPV1 has only GP 33–41 inactivated but retains CTL epitopes GP 276–286, GP 92–101 and GP 118–125. Five to six mice formed each experimental group, and duplicate experiments had similar results. Panel A: wt LCMV (ARM) and the two variants GPV1 and GPV replicated equivalently over time after infection of H-2^b^ C57Bl/6 MC-57 fibroblasts or H-2^d^ Balb Cl-7 fibroblasts. Culture supernatants were obtained at the times shown, centrifuged free of cells, plaqued on Vero cells and tested in triplicate samples. Panel B: intracellular IFN-γ in virus-specific splenic lymphocytes 7 days post-infection with wt LCMV or the LCMV variants GPV1 and GPV. Flow data from two of six mice are shown along with mean values ± SE of six mice per group for CD8 CTL epitopes GP 33, GP 276, GP 92, GP 118, NP 396 and the CD4 T cell epitope GP 67. Analyses performed 15 days after the initiation of virus infections yielded similar results. Data are representative of two independent experiments.

Initial studies using a single-cycle growth curve (Fig. 1A) indicated that wild-type (wt) LCMV, GPV1 (loss of GP 33 epitope only) and GPV (loss of GP 33 and GP 276 epitopes) generated equivalent numbers of viral progeny when used at similar multiplicities to infect Vero indicator cells. The three viruses replicated to similar extents at 24, 48, and 72 hours (data not shown for 72 hours). Inoculation with 1×10^5^ PFU of each virus, using four to five mice/group, generated CD8 or CD4 virus-specific T cells with the expected specificities (Table 1, Fig. 1B). Thus, as shown in Table 1, wt LCMV ARM and the two variant GPV1 and GPV, when inoculated into H-2^b^ mice, generated virus-specific CTL to H-2^b^-restricted targets but not to H-2^d^ targets and showed robust CTL responses to a vaccinia virus (VV) recombinant expressing the immunodominant NP epitope (NP 396–404). In terms of recognizing GP epitopes, wt ARM generated robust CTL that recognized VV expressing whole GP or GP1 (all LCMV GP except the GP 33–41 epitope). By contrast, CTL generated by GPV1 inoculation recognized VV expressing whole GP but not VV expressing GP1 minus the GP 33 epitope. CTL generated following inoculation of GPV responded strongly to the nucleoprotein (NP) epitope expressed in H-2^b^ cells infected with wt LCMV or VV recombinant expressing LCMV NP but responded negligibly to the VV recombinant expressing whole GP or GP missing the GP 33 epitope. When mice were inoculated with the various viruses, wt LCMV (ARM) generated an average of 12.5±2.0% CD8 T cells to GP33, 4.1±0.6% to GP 276, <1% to GP92, and 2.3±0.1% to GP118 (Fig. 1B). In total, 20% of all CD8 T cells were specific for LCMV GP. By contrast, GPV1-infected mice showed a significant reduction in GP33-specific CD8 T cells, but no significant change in the generation of cells specific to the remaining CD8 T cell epitopes: GP276, GP92, or GP118. When GPV was used to challenge mice, there was a significant reduction in the percentage of GP33 (96%) and GP276 (98%) specific CD8 T cells but no significant reduction in the percentage of cells directed against subdominant GP92 and GP118 CD8 T cell epitopes (Fig. 1B). As expected, inoculation with LCMV GPV1 or GPV had a negligible effect on the generation of immunodominant NP396-specific CD8 T cells or GP67-specific CD4 T cells (Fig. 1B) [24], [26], [27].

**Table 1:** CTL specificity of wt and LCMV variants used.

% specific ^51^Cr released from targets infected with	
effector CTL from day 7 P° spleen		H-2^b^				H-2^d^
VV recombinant
H-2 mice	1×10^5^ PFU virus ip	LCMV ARM	NP	GP	GP1	LCMV ARM
H-2^b^	ARM	57±11	34±2	32±5	32±8	2±2
H-2^b^	GPV1	57±5	31±4	43±5	3±1	1±1
H-2^b^	GPV	49±8	40±8	2±1	1±1	2±2
H-2^b^	ARM	3±2	0	3±2	5±1	51±10

LCMV variants GPV1 or GPV, or the parental wt LCMV ARM virus, were inoculated (1×10^5^ PFU i.p.) into C57Bl/6 (H-2b) or Balb/cdj (H-2d) mice. Seven days later lymphocytes were obtained from spleens and used in a cytotoxic ^51^Cr-release assay at an effector to target ratio of 50∶1 as described in Materials and Methods. The generation, selection, sequencing and specificity of GPV1 and GPV have been published [26], [27]. Target cells H-2^b^ (MC-57) and H-2^d^ (Balb Cl 7) as described [25], [26], [50] were infected with LCMV ARM or VV constructs expressing the whole wt LCMV nucleoprotein (NP), whole LCMV GP, or just the N-terminal component of GP containing the GP-33 CTL epitope but not the GP-276 CTL epitope [50]. Numbers represent the mean of triplicate samples ± 1 SD from four mice per experimental group.

**Figure 2:**
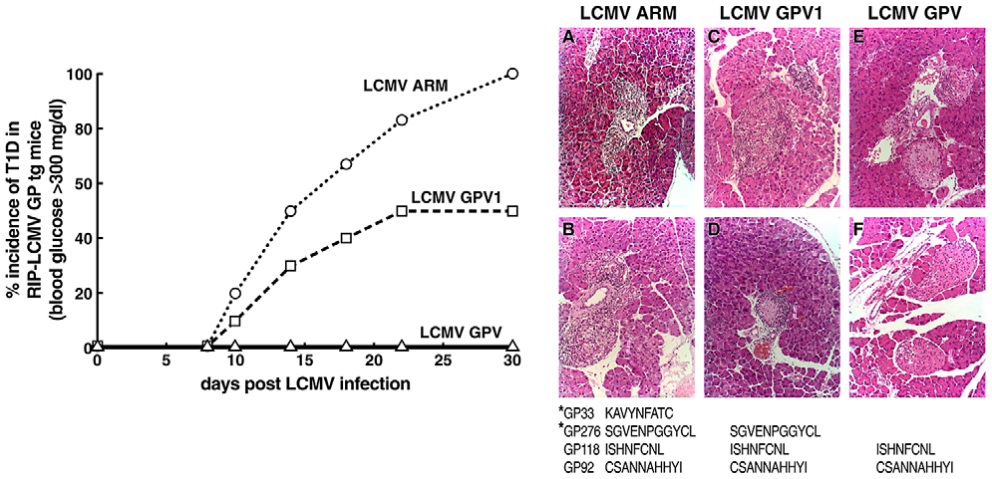
Induction of T1D with wt LCMV ARM and its GPV1 or GPV variants. RIP LCMV GP tg mice were inoculated i.p. with 1×10^5^ PFU of either wt LCMV ARM, GPV or GPV1. Left panel: incidence of T1D over time for virus-inoculated mice. Right panel: histologic profiles of islets from mice infected with each viral strain. Tissue was taken when mice had blood glucose of 300 mg/dl or more (right panels A, B, C) or blood glucose below 200 mg/dl at day 30 (right panels D, E, F). Eight to ten mice per experimental group. Experiments repeated once with similar results.

We next determined the ability of wt LCMV ARM, GPV1 and GPV viruses to cause T1D in C57Bl/6 (B6) RIP-LCMV GP mice. As anticipated from our prior reports [12], [25], [28], [29], inoculation of RIP-LCMV GP tg mice with 1×10^5^ PFU of LCMV ARM i.p. induced T1D in all mice (Fig. 2, left panel). By four weeks post-infection with wt LCMV ARM, all mice had blood glucose levels of >300 mg/dl, massive cellular lymphocyte infiltration in their islets of Langerhans (Fig. 2, right panel A, B). In contrast, all seven age- and sex-matched RIP-LCMV tg mice not inoculated with virus and all five B6 mice inoculated with 1×10^5^ PFU of LCMV i.p. had blood glucose levels <200 mg/dl, and no lymphocytic infiltrates in their islets (data not shown).

To evaluate the ability of the LCMV variants to cause T1D, we inoculated each of nine RIP-LCMV GP tg mice with 1×10^5^ of LCMV GPV i.p., i.e., the variant in which both H-2^b^ immunodominant GP33 and GP276 epitopes are knocked-out but the two subdominant GP CTL epitopes GP92 and GP118 remain. T1D was not observed in any of the mice (Fig. 2, left panel), and minimal to negligible lymphoid cells infiltration in the islets (Fig. 2, right, panels E, F). From these results, we conclude that T1D does not occur when a CTL response is directed against subdominant GP CTL epitopes (GP92 and GP118) but does to the immunodominant GP CTL epitopes (GP33 and GP276). Interestingly, although T1D was not caused by the remaining GP virus-specific CD8 T cells (5%) generated in the spleen (Fig. 2), subdominant GP-specific CD8 T cell responses are sufficient to protect the immunized mice from an acute LCMV infection [27], [30]. The immunodominant CD4 epitope GP 67–80 within LCMV GPV generated anti-LCMV CD4 T cells (Fig. 1B), those virus-specific CD4 T cells failed to help the few (5%) LCMV-specific GP CD8 CTLs present in the spleens of GPV-infected mice to cause T1D. By contrast, when RIP-LCMV GP tg mice were infected with GPV1 (containing the mutated GP33 CTL epitope) the T1D incidence was reduced to 50% (Fig. 2, left panel) and was accompanied by a moderate lymphoid cell infiltration in the islets of those developing diabetes (Fig. 2, right, panels C, D). In some recipients of GPV1 that did not develop T1D, the lymphoid cell infiltrate cuffed around the periphery of the islets (Fig. 2, right, panel D). Other mice infected with GPV1 but not developing diabetes showed only a modest lymphoid infiltration in the islets. These results indicate that a threshold of virus-specific CD8 T cells is required to cause T1D. Twenty-percent of total generated virus-specific CD8 T cells following wt LCMV infection are sufficient to cause T1D in all mice, but when percentage of virus (self) reactive CD8 T cells was reduced to 9%, only 50% of mice developed T1D, and when such numbers were further reduced to 5%, T1D did not occur. Further, these results clearly demonstrate that a threshold of specific antiviral (self) CD8 T cells are required to cause diabetes and suggest that a reduction of virus (self)-specific CD8 T cells below that threshold will prevent T1D. Thus, T1D is quantifiable and complete elimination of effector cells is not required to abort disease.

### Quantification of immunodominant LCMV-specific GP33 CD8 T cells in the islets of Langerhans during development of T1D

**Figure 3:**
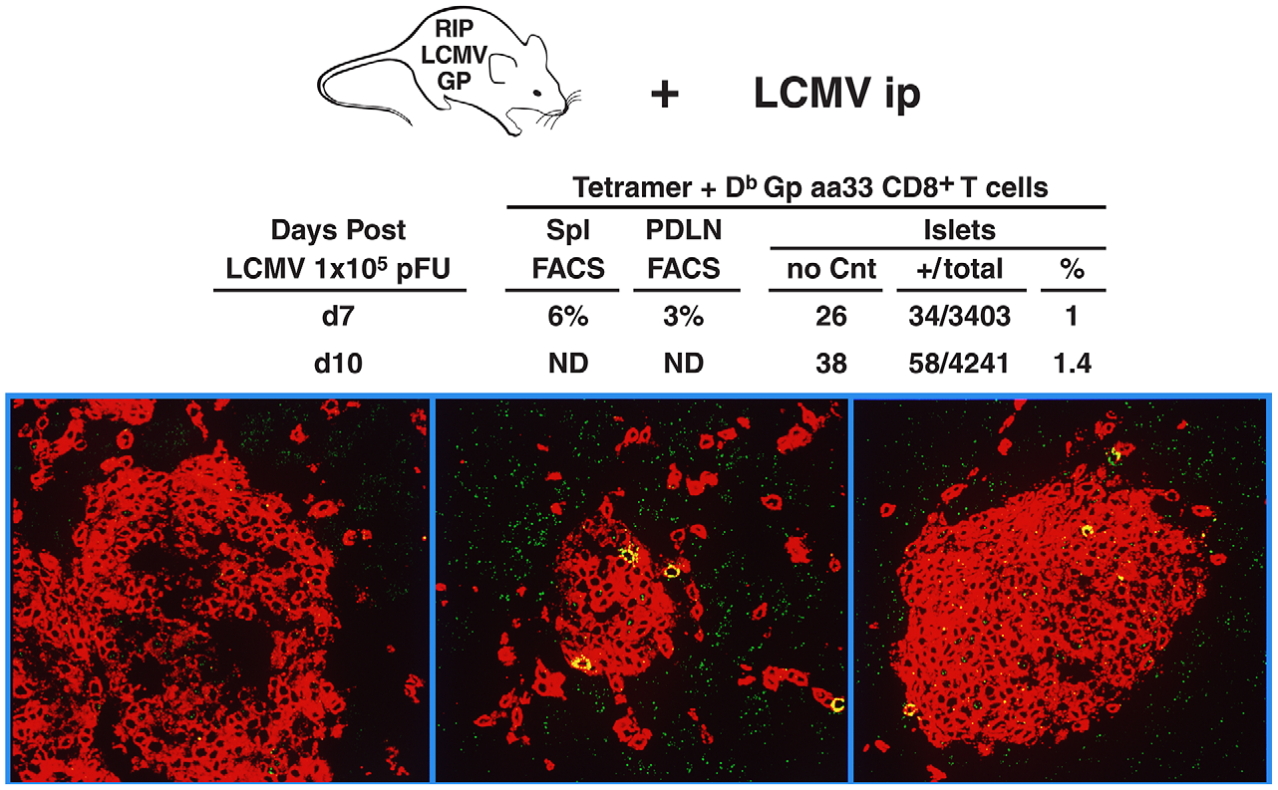
Quantification of virus-specific CD8 T cells from bystander non-virus-specific CD8 T cells in the islets of RIP LCMV GP mice during the induction of T1D. Seven or ten days after RIP LCMV GP mice were injected i.p. with 1×10^5^ PFU LCMV ARM, sections of their pancreases were stained with tetramer *in situ* and viewed by confocal microscopy. Red fluorochrome dye marks all CD8 CTL in the islets reactive with antibody to CD8. Yellow represents the virus-specific CD8 T cells (tetramer positive) examined by FITC (double-labeling with CD8 and tetramer). Numbers were calculated by sampling multiple islets from four infected mice (see Materials and Methods and [31] for details).

To quantify the numbers of virus (self)-specific CD8 T cells that migrate into the islets during T1D, we inoculated RIP-LCMV GP mice with 1×10^5^ PFU LCMV ARM i.p. and harvested their pancreata at 7 and 10 days post-infection. Six micron sections cut by cryomicrotome were then stained with D^b^ GP 33–41 tetramer. Figure 3 shows the massive CD8^+^ T cell infiltrate in the islets visible by using a CD8 monoclonal antibody labeled with red fluorochrome. Surprisingly, when pancreas sections were co-stained with D^b^ GP 33–41 tetramers, an exceedingly small number (1–2%) of the infiltrating virus-specific CD8 T cells were determined to be GP33-specific (Fig. 3). These numbers were similar for islets studied at days 7 and 10 post-infection. Of 26 islets studied at day 7, 1% (34/3403) of the CD8 T cells were GP33-specific. Similarly, 1.4% (58/4241 cells from 38 islets) were GP33-specific at 10 days post-infection. Earlier studies determined that the fidelity of this *in situ* tissue tetramer technique compared favorably to that of FACS [31]. For those studies GP33 T cell receptor (TCR) tg mice were sacrificed, spleens removed and virus-specific CD8 T cell numbers quantified by flow cytometry versus in situ GP33 tetramer staining in the same spleen. Ninety-two percent of CD8 T cells from the spleens of naïve GP33 TCR tg mice were positive by FACS compared to 86% (84–90%) by *in situ* staining. Thus, the error in the in situ tetramer assay is no more than 5%, which is unable to account for the low numbers of virus (self) specific GP-33 CTL among the massive influx of non-virus-specific bystander CD8 T cells. Figure 3 also depicts the numbers of virus-specific GP 33 CD8 T cells in the spleen and pancreatic draining lymph nodes as well as the islets in RIP-LCMV tg mice 7 and 10 days post-infection. In summary, these results indicate that although virus-specific CD8 T cells are absolutely essential in causing diabetes, only 1–2% of the GP33 CD8 T cells in the islet target cells are involved. Thus, of the 12.5% GP33 T cells generated in the spleen, fewer than 10% of the total are found in the islets.

### Probing the immunologic synapse between CD8 T cells and pancreatic β cells

**Figure 4:**
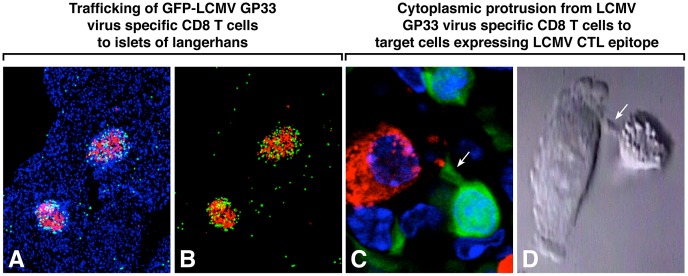
Analysis of virus specific CD8 T cells in the islets of Langerhans. GFP-labeled GP-33 LCMV-specific CD8 T cells were adoptively transferred into RIP-LCMV GP tg mice. Three days later mice, received 1×10^3^ PFU of LCMV ARM (see Materials and Methods). Panels A and B: trafficking of GFP (green)-labeled GP-33 CD8 CTL to islets (red represents staining by antibody to insulin). Panel C: higher power of cytoplasmic protrusion (proboscis, note arrow) from GFP-stained GP-33 specific CD8 CTL reaching out to β cell (insulin stained red). Panel D: similar cytoplasmic protrusion (arrow) seen *in vitro* when GP-33 LCMV is incubated with LCMV-infected MHC-restricted H-2b fibroblasts [32]. Note granules containing perforin and other serine esterases in the cytoplasm of the CD8 CTL.

**Figure 5:**
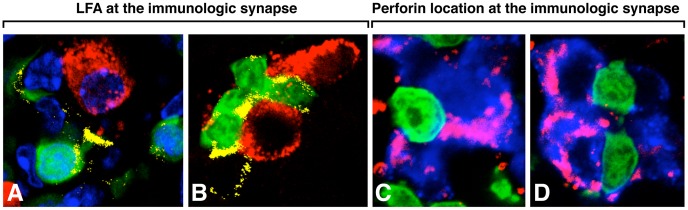
Detection of LFA-1 and perforin at the immunologic synapse between virus-specific CD8 CTLs and β cells in the islets of Langerhans. Virus-specific GP-33 CTL stained with GFP were adoptively transferred into RIP-LCMV GP tg mice. In panels A and B, the position of LFA-1 molecules (yellow) in the context of GFP CTL and β cells (insulin labeled red) is shown. Panel A, note cytoplasmic protrusion (proboscis) and LFA stain (yellow) at the point of contact with the β cell. Panels C and D show release of perforin (pink) in the cytoplasm of target β cells (marked blue for insulin stain). The CTL are marked in green. See Materials and Methods and reference [31] for details.

To visualize selected molecules in the immunologic synapse during development of T1D, we tracked adoptively transferred virus-specific GP33 CD8 T cells genetically tagged with GFP [31]. 10^4^ GFP^+^ CD8^+^ D^b^ GP33–41-specific T cells were adoptively transferred intravenously (i.v.) into RIP-LCMV GP tg mice. Three days later, these mice received 1×10^5^ PFU of LCMV ARM that resulted in a >10^4^-fold expansion of GFP-labeled virus-specific CTL in the spleen five days later [31]. Afterward, these GFP^+^ CD8^+^ D^b^ GP33–41-specific T cells migrated to the islets of Langerhans (Fig. 4, panels A, B) and induced T1D as early as 9–10 days post-LCMV challenge. Illustrations of these GFP^+^ CD8^+^ Db GP 33–41 T cells anatomically interacting with β cells of the islets are displayed in Figure 4, panel C, and Figure 5. Note the cytoplasmic extension of the virus-specific CD8 T cell (green) reaching to make contact with β cells (insulin labeled with red) (Fig. 4C arrow). This picture observed *in vivo* is reminiscent of our earlier in vitro studies of cloned LCMV-specific CD8 T cells interacting with a D^b^ LCMV-infected fibroblast *in vitro* (Fig. 4D arrow) [32].

We then used antibodies specific to LFA-1 or perforin to visualize these molecules at the immunologic synapse in the islets of mice developing T1D. Both molecules were found at the interactions between D^b^ GP 33–41 GFP^+^ CD8 T cells and β cells (Fig. 5). The integrin LFA-1 marked in yellow (Fig. 5, panels A, B) allowed the virus-specific CD8 T cell (green) to engage the target β cell (red). Perforin (pink) was deposited on β cells (blue, stained for insulin) by β cell virus-specific CD8 T cells (green) suggestive of impending destruction (Fig. 5, panels C, D). Perforin, or pore-forming protein, is present in the lytic granules of CTL displayed in Fig. 4, panel D and is polarized towards the target cell after T cell engagement.

## Discussion

We developed a tg mouse model where a viral transgene, the LCMV full-length GP, was expressed only in β cells of the pancreatic islets of Langerhans [25]. While insertion and expression of the viral gene, *per se* did not lead to diabetes (incidence <1%), the induction of an anti-LCMV CD8^+^-specific CTL response that reacted with the viral expressed transgene effectively and routinely produced T1D (incidence >94%). Previous studies indicated that CD4 T cells failed to play a role in this model, since their depletion did not alter either the kinetics or incidence of the resultant diabetic disease [12], [28]. This result stands in contrast to a model in which the viral transgene was expressed both in the β cells and in the thymus. In this model, anti-LCMV CD8 T cells of high affinity are selected out in the thymus with antiviral CD8 T cells of lower affinity going to the periphery. To cause T1D, virus-induced specific CD4 T cells in addition to virus-specific CD8 T cells were required [12], [28], [33].

The full-length viral GP in H-2^b^ mice has only four distinct CD8 CTL epitopes that recognize two immunodominant LCMV epitopes, GP33 and GP276, as well as two weaker subdominant CD8 CTL epitopes, GP92 and GP118. Deletion of both immunodominant GP epitopes from LCMV (GPV) failed to cause T1D despite the generation of virus-specific CD8 CTL to the two weak subdominant GP epitopes. The percentages of GP-specific CD8 CTL generated in the spleen following infection with GPV were as follows: GP33 (1.2%), GP276 (0.1%), GP92 (<1%), and GP118 (2.7%) for a total of 5% as determined by tetramer analysis. This number of virus-specific CD8 T cells was insufficient to cause diabetes. Islets in RIP-LCMV GP tg mice infected with GPV routinely failed to show an accumulation of T cells; there was no β cell damage, and blood glucose levels were normal. Although virus-specific CD4 T cells were generated, they were inefficient in providing help to virus-specific CD8 T cells to damage the islets or incite T1D. In contrast, when the total percentage of LCMV GP-specific CTL in the spleen reached 20%, diabetes was observed in >94% of RIP-LCMV GP tg mice, and if the percentage was 9%, T1D occurred in 50% of RIP-LCMV GP tg mice. These findings indicate that there is a quantifiable number of LCMV GP CD8 T cells required to cause diabetes and that a therapeutic approach directed to lowering or limiting; but not necessarily eliminating virus (self) specific CD8 T cells is likely to be successful in treating T1D.

Following LCMV infection, 12.5% of CD8 T cells in the spleen are specific to the dominant GP33 epitope which leads to migration of these virus-specific CD8 T cells into the islets. Unexpectedly, only a small number of these virus-specific CD8 CTL, on the order of 1–2% of the total virus-specific CD8^+^ T cell population were found in the islets during the peak of disease. Earlier studies in the LCMV model indicated that, at any one snapshot in time, a single CTL could engage up to three target cells *in vivo* [31], and under optimal in vitro culture conditions, one virus-specific CD8 CTL could interact and lyse up to 10–12 virus-infected target cells [32]. Thus, it is likely that in vivo CTLs amplify their efficiency in killing β cells by engaging and moving to multiple targets. Nevertheless, since only a low number of effector cells are required to cause disease, a therapeutic focus on reducing the numbers of such cells may be sufficient to curtail β cell damage and prevent T1D. Indeed, past studies using specifically designed peptides that competed for MHC binding to immunodominant LCMV CD8 T cells were able to diminish or abort diabetes [34]–[36]. Additionally, the number of effector CD8 T cells, as documented in Listeria infection, appears tightly controlled by TGF-β-mediated apoptosis [37]. This suggests that TGF-β and likely other factors may also play a role in our in vivo analysis of the generation of effector CD8 T cells.

Analysis of interactions at the immunologic synapse between virus-specific CTL and β cell targets following in vivo infection showed a proboscis or tongue-like extension from the CTL cytoplasm to the target cell (Figures 4 and 5). This CTL extension appears anchored by LFA molecules that connect the target cell to the killer cell and provide a bridge for the passage of cytotoxic granules onto and into the β cell. Indeed, in vitro studies clearly showed that cytoplasmic granules containing serine esterases travel down that proboscis-like bridge to the target cell. This leads to a puncturing of the target cell membrane followed by actin/myosin dissociation, blebbing, and cellular destruction [32]. The binding of virus-specific CD8 T cells to β cells and the demonstration of perforin-like molecules released by CTL onto β cells strongly suggest a lytic role for CD8 T cells in destroying β cells to cause T1D. In addition, inflammatory cytokines like interferon-γ generated by virus-specific CD8 T cells are important contributors to T1D [38]. Interestingly, in LCMV-induced acute leptomeningitis, LCMV-specific CD8 T cells caused damage indirectly by first identifying the virus-infected target followed by a release of cytokines and chemokines which call in myeloid cells that also participate in cellular injury [39]. Whether a similar event occurs or plays a role in virus-induced diabetes is unknown.

While our data clearly demonstrate that an overlap between a viral and self-protein can give rise to T1D following infection, others have suggested that bystander T cell activation might also serve as a disease trigger [40]. Bystander activation occurs when an infection causes T cells to acquire effector functions in a non-specific manner [41]. Infection of BDC2.5 T cell receptor transgenic mice with coxsackie virus CB4 strain (a pancreatrophic virus) results in a 90% incidence of T1D [40]. Because BDC2.5^+^ CD4^+^ T cells recognize an islet antigen, but do not cross-react with coxsackie virus, it was concluded that T1D was induced by bystander activation of self-reactive T cells. It is important to note, however, that bystander T cell activation is a relatively inefficient process estimated to occur in only 1 in 200 virus-specific CD8^+^ T cells infected with a non-overlapping pathogen [42]. Thus, the high frequency of islet-specific T cells in the repertoire of BDC2.5 mice likely favors the induction of T1D following coxsackie virus infection [40]. Importantly, when non-transgenic diabetes-prone NOD mice were infected with coxsackie virus, the pathogen could accelerate but not initiate T1D [40], [43]. These data suggest that viruses may accelerate autoimmune diseases through bystander activation once a critical threshold of self-reactive T cells is reached. A shared epitope between a viral and self-protein (molecular mimic) is presently the most efficient mechanism to generate the number of self-reactive T cells required for an autoimmune disease to develop, and once initiated, a pathogenic disease process does not need to rely on bystander T cells [44], [45], although they may contribute to tissue pathology [46]. With the low levels of virus-specific T cells in the islets it is unlikely that bystander T cells play a role in T1D in our model.

Host genes, autoimmune responses, cytokines/chemokines and virus have all been implicated in the initiation and progression of T1D. In our view, these various components and activities can be integrated into one theme associated with two distinct events. First, a viral or other environmental exposure occurs early in life and results in restricted but low level expression of the viral or microbial gene(s) in β cells of the islets of Langerhans. This is an event that by itself need not cause disease since most hosts are hyporesponsive or tolerant to the viral product. Later, a second event occurs. Infection with the same virus (or microbe) or one bearing cross-reacting antigenic determinants or conformation shapes lead to an immune response that activates sufficient CD8 T cells to migrate to and infiltrate pancreatic islets, injure β cells, and cause T1D. Manipulations that lower the numbers or functions of activated CTL or impair their migration to the islets should be beneficial as therapy for T1D.

## Materials and Methods

### Ethics statement

Animal care and breeding are performed in the AAALAC accredited vivarium (Vertebrate Animal Assurance No. A3194-01) at The Scripps Research Institute. All aspects of mouse experimentation follow the guidelines in the NIH Guide for the Care and Use of Laboratory Animals and are approved by TSRI Animal Use Committee.

### Tg cell lines, mice, and viruses

Generation, breeding, genotyping and characterization of the tg cell line RIP GP-34 LCMV has been detailed [25], [28]. These mice were bred onto the C57Bl/6 (H-2^b^) background for over 25 generations.

LCMV ARM Clone 53b and its GPV1 and GPV variants were used for this study [26], [27]. These viruses replicate equivalently (Fig. 1), cause acute infection, and generate virus-specific CD8 CTL. Escape CTL variants for GP 33–41 (GPV1) and GP 276–286 (GP2V) and for GP 33–41 plus GP 276–286 (GPV) were selected [26], [27] and characterized by single amino acid substitutions for the GP33 epitope at aa 38 with a UUU→UUG (F to L) change and for GP276 epitope at aa 282 a GGU→GAU (G to D) substitution. These substitutions resulted in the reduction of CD8^+^ CTL killing of H-2^b^ targets expressing either GP 33–41 or GP 276–282 or GP 31–41 plus GP 276–286 epitopes or peptides, respectively. The two variants (GPV1 and GPV) had equivalent binding to MHC H-2^b^ antigen-presenting cells. In addition, wt LCMV, GPV1 and GPV contained the subdominant GP 92–101, GP 118–125 and immunodominant nucleoprotein (NP) 396–404 CD8 CTL epitopes and the immunodominant CD4 CTL epitope GP 67–80 [24], [26], [47], [48]. LCMV wt, GPV1 and LCMV GPV displayed normal migration *in vivo* [27].

### ^51^Chromium (Cr) CTL assay

The ^51^Cr-release assay was done 7 days after inoculating C57Bl/6 (H-2^b^) mice or Balb/cdj (H-2^d^) mice i.p. with 1×10^5^ PFU of virus [25], [26]. Single-cell suspensions of lymphoid cells harvested from spleens of mice infected with wt LCMV, LCMV GPV1 or LCMV GPV were added at effector-to-target ratios of 50∶1 and 25∶1 to H-2^b^ (MC57) or H-2^d^ (Balb Cl7) target cells. Target cells were pre-labeled with ^51^Cr and infected with LCMV ARM or various vaccinia virus (VV) constructs expressing LCMV GP components [25], [26], [49], [50]. We used VV expressing whole LCMV GP (VV GP), VV expressing only the LCMV GP1 epitope (aa 33–41), (VV GP1), or VV expressing only the LCMV NP (VV NP). Construction of recombinant VV with LCMV GP, LCMV GP1 or LCMV NP followed by report of Mackett et al. [51], and was confirmed by Northern blot analysis of LCMV-specific RNA [50]. For each, study, groups of four to five mice were infected, and their CTL profiles were investigated on three independent samples obtained from each mouse. The results are presented as means ± 1 SD of the independent samples. Experiments repeated two times with equivalent results.

### Blood glucose

Glucose was measured in blood samples obtained from the retro orbital plexus of each mouse before administering virus or vehicle (day 0) and then at days 5, 8, 10, 14, 18, 22, and 30 post-virus inoculation. Plasma glucose concentrations were determined using One-Touch Ultra (LifeScan, Inc., Milpitas, CA, USA) [29].

### Histology and tetramer staining *in situ*

Tissues taken for histological analysis were fixed in zinc formalin (10%) and stained with hematoxylin and eosin. Immunochemical studies were carried out on 6–10 µm cryomicrotome-sliced sections , handled, stained and fixed as described [23], [25], [29], [31]. Briefly, each pancreas was removed and quick-frozen in O.C.T. compound (Tissue-Tek, Torrance, CA, USA) on dry ice. Cryomicrotome sections were incubated overnight at 4°C in 2% fetal calf serum (FCS) containing MHC class I LCMV tetramers GP 33–41 conjugated to allophycocyanin (0.2 µg/ml) and rat anti-mouse CD8 (0.2 µg/ml; Pharmingen, San Diego, CA, USA). Tissues were then fixed with 2% formaldehyde, washed and incubated at 4°C for 3 hr with rabbit antibody to allophycocyanin (1∶1000 dilution, Biomeda, Hayward, CA, USA) then washed and incubated at 4°C for 3 hr with Cy5-rabbit antibody (1∶500) and Rhodamine Red X-antibody (1∶500).

### Peptide stimulation and tetramer staining

Quantification of virus-specific T cells and functional analysis of their ability to express the inflammatory cytokines IFN-γ and TNF-α or display IL-2 in their cytoplasm were performed using our established and published assay for the T cell epitopes of LCMV ARM [47], [52], [53]. Briefly, for intracellular cytokine analysis and flow cytometry, splenocytes were stimulated with 2 µg/ml of MHC class I-restricted GP 33–41, GP 276–286, or 5 µl of MHC class II-restricted GP 67–80 peptide (>99% pure) in the presence of 50 U/ml recombinant murine IL-2 and 1 mg/ml Brefeldin A. Cells were stained for surface expression of CD8 (clone 53-6.7, Caltag), fixed, permeabilized and stained with antibodies of IFN-γ (clone XMG1.2), TNF-α (clone MPG-XT22) or IL-2 (clone JES6-5H4) from Pharmingen. Flow cytometric analysis was performed using a digital LSR-II (Beckman). The absolute number of virus-specific T cells was determined by multiplying the frequency of tetramer or IFN-γ+ cells by the total number of cells in the spleen.

### Immunochemical analysis of LCMV GP33-specific CTL engagement of β cells *in vivo*

CD8 T cells specific for LCMV GP 33–41 epitope were purified from GP33 TCR-tg×β-actin expressing GFP tg F_1_ mouse splenocytes by negative selection (Stem Cell Technologies, Vancouver, Canada) as described [31]. More than 98% of enriched cells were CD8^+^, and 80% of the cells were double-positive for Db-GP 33–41 tetramers and GFP. 1×10^4^ double-positive cells were transferred intravenously into naïve 6- to 8-week-old RIP-LCMV GP tg mice. Three days later the mice received an intraperitoneal (i.p.) injection of 1×10^5^ PFU of LCMV or its various variants. For analysis of LFA-1 and perforin on GFP^+^ CTL and the β cell targets (identified by containing insulin), the pancreas was sectioned, and rat anti-LFA-1 (3 µg/ml; Pharmingen), goat anti-perforin (2 µg/ml, Santa Cruz Biotechnology, Santa Cruz, CA, USA) or anti-insulin antibody was added. Primary antibodies to LFA-1, perforin and insulin were labeled with the appropriate secondary antibodies conjugated to Cy5 as described [31]. Three-color (GFP, Rhodamine Red-X and Cy5) analyses were done to visualize cell-cell interactions. Four-color (DAPI, GFP, Rhodamine Red-X and Cy5) 3D datasets were collected with a DeltaVision system (Applied Precision, Issaquah, WA, USA); this consisted of an Olympus IX-70 fluorescence microscope, a motorized high-precision *xyz* stage, a 100-W mercury lamp and KAF1400 chip-based cooled charge-coupled device camera. Exposure times were 0.1–0.8 s and images were obtained with a ×100 oil objective.
